# Should chloroquine and hydroxychloroquine be used to treat COVID-19? A rapid review

**DOI:** 10.3399/bjgpopen20X101069

**Published:** 2020-04-08

**Authors:** Kome Gbinigie, Kerstin Frie

**Affiliations:** 1 GP and Doctoral Researcher, Nuffield Department of Primary Care Health Sciences, University of Oxford, Oxford, UK; 2 Post-Doctoral Researcher, Nuffield Department of Primary Care Health Sciences, University of Oxford, Oxford, UK

**Keywords:** COVID-19, coronavirus, Chloroquine, Hydroxychloroquine, primary healthcare, general practice

## Abstract

**Background:**

On the 11 March 2020, the World Health Organization (WHO) declared that COVID-19 was a pandemic. To date, there are no medical treatments for COVID-19 with proven effectiveness. Novel treatments and/or vaccines will take time to be developed and distributed to patients. In light of this, there has been growing interest in the use of existing medications, such as chloroquine (CQ) and hydroxychloroquine (HCQ), as potential treatments of this disease.

**Aim:**

To establish the current evidence for the effectiveness of CQ and HCQ in treating COVID-19.

**Design & setting:**

A rapid review of the literature was conducted.

**Method:**

Electronic searches in PubMed and Google Scholar were conducted on 21 March 2020. A further search was conducted in Google for relevant literature on 28 March 2020.

**Results:**

There is limited evidence of in vitro activity of CQ/HCQ against SARS-CoV-2. A number of in vivo clinical trials are underway. The empirical data available from two of these trials reveal conflicting results. Both trials are characterised by small numbers of participants (*n* = 30 and *n* = 36) and suffer methodological limitations. No medium or long-term follow-up data is available.

**Conclusion:**

At present, there is insufficient evidence to determine whether CQ/HCQ are safe and effective treatments for COVID-19. High quality, adequately powered randomised clinical trials in primary and secondary care settings are urgently required to guide policymakers and clinicians. These studies should report medium- and long-term follow-up results, and safety data.

## How this fits in

Chloroquine (CQ) and hydroxychloroquine (HCQ) have been used in the treatment and prophylaxis of a number of conditions, such as malaria, for several years. As novel treatments for COVID-19 are likely to take time to develop, a number of clinical trials have been registered to investigate the effectiveness of existing medications such as CQ or HCQ. At present, there is insufficient evidence to recommend their use for the current pandemic outside of clinical trials. Further, high quality studies are urgently needed to provide timely guidance for clinicians and policymakers alike.

## Introduction

To date, there have been over 17 000 cases and 1 000 deaths due to COVID-19 in the UK alone.^1^ There are intensive efforts worldwide to develop novel treatments for COVID-19 as the pandemic continues to spread. Such treatments will take time to develop and become available for widespread use. In the interim, there has been increasing interest in the use of existing medications to treat COVID-19, including CQ and HCQ. CQ was first used as prophylaxis and treatment for malaria. HCQ is a more soluble and less toxic metabolite of CQ. It causes fewer side effects and is therefore considered safer than CQ.^2–4^

The antiviral properties of both medications have been researched in recent years. CQ and HCQ have been used in the treatment of HIV with mixed results.^5^ The ability of these drugs to inhibit other coronaviruses, such as SARS-CoV-1, has been explored with promising results.^6,7^ There are a number of theories to explain the potential mechanism of action of CQ/HCQ against SARS-CoV-2. The virus is believed to upregulate cell surface angiotensin converting enzyme 2 (ACE2),^8^ binding to this enzyme in order to facilitate host cell entry.^9^ CQ may reduce glycosylation of ACE2, thus preventing this attachment process.^10^ Another potential mechanism involves the inhibition of viral release into the intracellular space. Within the host cell, the virus is surrounded by a cell-membrane derived vesicle — the endosome — within which the virus replicates.^11^ CQ is believed to accumulate in lysosomes, which may interrupt the usual process of lysosome–endosome fusion, thereby inhibiting release of the viral contents.^11,12^ Moreover, CQ may also block the production of interleukin-6 and other pro-inflammatory cytokines, which are key mediators of acute respiratory distress syndrome (ARDS).^11^

Both drugs are affordable, widely available internationally, and are generally considered safe for their present US Food and Drug (FDA)-approved indications. However, these drugs have been incorporated into some national guidelines to treat COVID-19 in certain situations, despite a lack of rigorous clinical trial evidence of effectiveness.^13,14^ Furthermore, while generally considered safe, there are potential risks associated with taking these medications. Across the world there have been several reports of overdoses in people self-medicating with CQ during the current pandemic.^15,16^ There is therefore an urgent need to determine the safety and efficacy of these drugs for treating COVID-19, in order to guide international efforts to contain the pandemic. The aim of this rapid review is therefore to provide a timely summary of the evidence to date for the safety and effectiveness of CQ/HCQ as COVID-19 treatments.

## Method

Electronic searches were conducted in PubMed and Google Scholar on 21 March 2020 using the search terms *chloroquine, coronavirus, SARS-Cov-2, 2019-NCov, and COVID-19. An additional search of Google was conducted on 28 March 2020 to find further relevant internet proceedings. Titles and abstracts of the search results were screened, and in vitro and in vivo studies assessing the use of CQ/HCQ for the treatment of SARS-CoV-2 were included. In addition, reviews of the existing literature on this topic were included. No time, language, or study type restrictions were applied. A Chinese native language speaker helped to translate Chinese articles. Inclusion criteria were deliberately broad, to maximise the capture of potentially relevant studies, including pre-prints. Exclusion criteria were not applied. This article provides narrative summary of the current evidence.

## Results

As described elsewhere, a limited number in vitro and in vivo studies have evaluated the use of CQ/HCQ in treating SARS-CoV-2 infection.^17^

### In vitro studies

There is some in vitro data supporting the ability of CQ and HCQ to inhibit SARS-CoV-2 activity.^2,18,19^ Wang *et al* found that CQ was highly selective in its activity against the virus rather than host cells.^19^ Liu *et al* ^18^ found a similar 50% cytotoxic concentration (CC50 — the concentration which results in 50% cell death) for CQ and HCQ, and found that CQ was more potent than HCQ against the virus. By contrast, Yao *et al* ^2^ found that HCQ was more potent against SARS-CoV-2.

### In vivo clinical trials

Very limited in vivo data assessing the effectiveness of CQ/HCQ for COVID-19 has been published to date (see Table 1). The first reports of clinical effectiveness of CQ for COVID-19 came from a news briefing by the Chinese government in February 2020. They reported a positive signal from the results of treating more than 100 patients with CQ in China, reportedly reducing the duration of illness, and improving COVID pneumonia and appearances on chest imaging.^20^ No adverse events were reported. These findings seem to be the combination of results from a number of ongoing clinical trials in different sites in China, using a variety of study protocols. No empirical data supporting these findings have been published thus far, and the evidence has therefore not been peer reviewed.

**Table 1.** Summary of characteristics of identified in vivo studies

**Authors (Year**)	**Country**	**Setting**	**Sample size (treatment/control**)	**Mean age, years (SD**)	**Inclusion criteria**	**Treatment**	**Primary outcome**	**Findings**
Reported by Gao **et al** (2020)^20^	China	10 hospitals in Wuhan, Jingzhou, Guangzhou, Beijing, Shanghai, Chongqing, and Ningbo	>100	Unknown	Tested positive for COVID-19, (unknown, potentially different across included trials)	Chloroquine phosphate	Unknown	*’* *Chloroquine phosphate is superior to the control treatment in inhibiting the exacerbation of pneumonia, improving lung imaging findings, promoting a virus-negative conversion, and shortening the disease course* *’*
Gautret **et al** (2020)^3^	France	Hospitals in Marseille, Nice, Avignon, and Briançon	36 (20/16)	Treatment: 51.2 (18.7)Control: 37.3 (24.0)	SARS-CoV-2 carriage in nasopharyngeal sample Age >12 years (treatment group only)	200 mg of Hydroxychloroquine three times a day for 10 days; six patients additionally received azithromycin	Outcome of a nasopharyngeal swab on Day 6	70.0% (treatment) versus 12.5% (control) virologically cured, *P*<0.001
Chen **et al** (2020)^21^	China	Shanghai Public Health Clinical Centre	30 (15/15)	Treatment: 50.5 (3.8)Control:46.7 (3.6)	Age ≥18 Tested positive for COVID-19 Hospitalised between 6–25 Feb 2020	400mg of hydroxychloroquine daily for 5 days	Outcome of a nasopharyngeal swab on Day 7	86.7% (treatment) versus 93.3% (control) virologically cured, *P*>0.05

The first empirical data from a clinical trial was published on the 6 March 2020 by Chen and colleagues from Shanghai, China.^21^ They conducted a randomised controlled trial to test the effectiveness of HCQ in 30 adult patients who tested positive for COVID-19. Patients in the treatment group received 400mg HCQ for 5 days, while the control group received usual care. The result of a nasopharyngeal swab on Day 7 was used as the primary outcome. The authors report three adverse events in the control group (one patient with abnormal liver function and anaemia, and one patient with abnormal renal function), and four adverse events in the treatment group (two patients with diarrhoea, one with lethargy, and one patient with abnormal liver function tests), which they argue were not linked to treatment with HCQ. One patient in the treatment group deteriorated significantly and thus HCQ was stopped on Day 4 of the treatment. The intention-to-treat analysis revealed that the treatment group did not differ from the control group in the number of patients testing negative for COVID-19 on Day 7 (13 versus 14 patients), nor the duration of illness (all *P*>0.05).

The second article reporting clinical trial data came from a French study published by Gautret and colleagues on 17 March 2020.^3^ The researchers conducted an open-label, non-randomised controlled trial with 36 patients diagnosed with COVID-19, of which six were asymptomatic, 22 had upper respiratory tract infection symptoms, and eight had lower respiratory tract infection symptoms. The twenty patients in the treatment group received HCQ 200mg three times a day for 10 days. Patients declining to take part in the study and not meeting the inclusion criteria were assigned to the control group and received usual care. Six of the patients in the treatment group additionally received azithromycin to prevent bacterial superinfection. The primary outcome was SARS-CoV-2 carriage at Day 6, detected from polymerase chain reaction (PCR) of SARS-CoV-2 ribonucleic acid (RNA) on nasopharyngeal swabs.

Patients in the treatment group were significantly more likely to test negative for SARS-CoV-2 on Day 6 than controls (70% versus 12.5% virologically cured, *P*<0.001). All six patients treated with HCQ and azithromycin tested negative on Day 6. The authors argue that these findings provide preliminary evidence of effectiveness of HCQ, and possible synergy between HCQ and azithromycin.

### Limitations of the current empirical data

The in vivo studies reported have methodological deficiencies. The trials by Chen *et al* and Gautret and colleagues were both underpowered according to their own calculations, which may have led to an exaggeration of effect sizes and false positive results.^22^ Moreover, whilst viral status at Day 6/Day 7 were the primary outcomes, no medium- or long-term follow-up data were presented in either study. Gautret and colleagues reported that one patient tested negative for the virus on Day 6, but subsequently tested positive on Day 8. This highlights the need for long-term follow-up data. Chen *et al* reported that most patients included in their trial (both control and treatment group) recovered quickly from COVID-19, indicating that the researchers had recruited a sample with mild symptoms. The authors argue that the lack of severe cases may have caused a ceiling effect, which may explain the non-significant results. Gautret *et al* ^3^ did not perform intention-to-treat analysis, excluding six patients from their analyses who had dropped out at follow-up, which may have introduced bias.^23^ Finally, Gautret and colleagues did not randomise patients to the control and treatment group, potentially introducing allocation bias.

## Discussion

### Summary

There is limited evidence suggesting in vitro activity of CQ/HCQ against SARS-CoV-2. The available in vivo empirical data is limited to two studies, with very small sample sizes, a number of methodological flaws, and conflicting results. On the basis of preliminary results from ongoing clinical trials, some countries have incorporated CQ/HCQ into their treatment protocols for certain patients with COVID-19. There is presently no medium to long-term follow-up data to support this approach. The very limited safety data available has not revealed serious side effects of these medications in the context of treating COVID-19.

### Strengths and limitations

To the authors’ knowledge, this is the first review that reports empirical data of the effectiveness of CQ/HCQ for treating COVID-19. By collaborating with a Chinese native language speaker, the authors were able to incorporate not only English articles, but also a Chinese report. This review was conducted quickly (10 days from searches being conducted to journal submission) in order to facilitate timely dissemination of the findings. As a result, however, these searches were not as rigorous as those of a systematic review. Some potentially relevant studies may therefore have been missed, particularly unpublished studies. The authors deliberately kept their search strategy broad to try to mitigate this and did not apply any exclusion criteria. Given the rapidly increasing number of patients infected with SARS-CoV-2 and the associated morbidity and mortality, the authors believe that the methodology employed was appropriate in this context.

### Comparison with existing literature

Two systematic reviews that have been conducted in this area were identified.^24,25^ Cortegiani *et al* ^24^ identified one in vitro study,^19^ a narrative letter reporting preliminary findings of several ongoing clinical trials in China,^20^ an editorial,^26^ an expert consensus paper,^27^ and two national guideline documents (Dutch and Italian). The review also provides an overview of the methodology of 23 ongoing clinical trials in China. The authors conclude that there are grounds to conduct high quality trials of CQ/HCQ for COVID-19. They did not report any empirical data for clinical trials of CQ/HCQ. A systematic review by Singh and colleagues^25^ identified two articles reporting the results of in vivo studies, which are also captured in this rapid review.^3,20^ Singh *et al* excluded studies written in Chinese, and therefore did not report the findings of the Chinese pilot study by Chen and colleagues.^21^ The present rapid review therefore serves as an important update, and reports more empirical data from in vivo studies than any other review on this topic, to the best of the authors’ knowledge.

There is limited safety data available for the use of CQ/HCQ in the context of COVID-19. In the report of over 100 patients in ongoing Chinese clinical trials presented in a news briefing by the Chinese government,^20^ no adverse events were reported. In the pilot study by Chen *et al*,^21^ adverse events related to gastrointestinal disturbance were only reported for the treatment group. However, the sample size makes it difficult to judge whether these side effects were caused by treatment with HCQ. Gautret and colleagues reported no adverse events, but stated that data on side effects would be published in a separate paper. CQ/HCQ are generally considered safe medications for their present FDAapproved indications;^10^ however, their prescription is not without risk. Gastrointestinal upset has been reported with HCQ.^28^ Retinal toxicity has been described with long-term use,^29,30^ and overdosage^30,31^ of CQ/HCQ. There have been isolated reports of cardiomyopathy^32^ and arrhythmia^33^ caused by treatment with CQ. CQ should be avoided in patients with porphyria.^34^ Both CQ and HCQ are metabolised in the liver with renal excretion of some metabolites, hence renal and liver function must be taken into account when these medications are prescribed.^34,35^ In a letter to the editor, Risambaf *et al* ^34^ raises concern about COVID-19 inducing liver and renal impairment, which may increase the risk of toxicity of CQ/HCQ when used to treat COVID-19. Patients’ comorbidities should be carefully considered before these drugs are prescribed.

### Implications for research and practice

Given the considerable limitations of the current available evidence, there is an urgent need for more, adequately powered, high quality randomised clinical trials to enable a better understanding of the effectiveness of CQ and HCQ for treating COVID-19. These studies should rigorously report safety data, as well as medium- and long-term follow-up data once this becomes available. Fortunately, more than 20 clinical trials are already registered to achieve this goal.^36^ These trials should help to inform the optimal dose and duration of treatment. All of the studies identified in this rapid review were conducted in secondary care. Studies in primary care settings are also needed to evaluate whether CQ/HCQ has utility in community settings. The authors are aware of one trial that has been registered to this purpose.^37^

The results of these studies should be made available in a timely fashion and to an international audience. This will facilitate synthesis of the results and enable important decisions to be made about the utility and safety of these drugs for the current COVID-19 pandemic. HCQ has a better safety profile, and may therefore be the preferred drug to focus initial research efforts on.

At present, there is insufficient evidence to recommend the prescription of these drugs by primary or secondary care physicians for COVID-19 outside of the context of research.

In conclusion, a limited number of in vitro studies report antiviral activity of CQ and HCQ against SARS-CoV-2. The data from in vivo studies is very limited, with mixed findings, which are likely to be prone to bias due to methodological limitations. Further research is now urgently needed in both primary and secondary care to determine whether CQ/HCQ are safe and effective treatments for COVID-19. At present, there is insufficient evidence to support their use in the management of COVID-19 outside of research.
